# The CD40 agonistic monoclonal antibody APX005M has potent immune stimulatory capabilities

**DOI:** 10.1186/2051-1426-3-S2-P198

**Published:** 2015-11-04

**Authors:** Pia Bjorck, Erin Filbert, Yongke Zhang, Xiaodong Yang, Ovid Trifan

**Affiliations:** 1 Apexigen, Inc., San Carlos, CA, USA

## 

The co-stimulatory receptor CD40 is a member of the tumor necrosis factor receptor (TNFR) superfamily and plays an important role in the control and regulation of immune activation, especially in crosstalk between T cells and antigen presenting cells (APCs). The natural ligand for CD40 (CD40L, CD154) is expressed on activated T cells and provides a major component of T cell “help” for the immune response. Agonistic CD40 antibodies can substitute for the function of CD154 on T cells to boost immunity by stimulating antigen presentation and co-stimulation to T cells and have been shown to be potent boosters of anti-tumor immune responses. Anti-CD40 can directly inhibit tumor growth in CD40 expressing tumor cells.

APX005M is a humanized IgG1 monoclonal antibody that binds CD40 with high affinity and blocks the binding of CD40 to CD40L. APX005M activates dendritic cells, B cells and monocytes, and promotes antigen-specific T cell responses. APX005M demonstrates potent anti-tumor activity via ADCC and induction of apoptosis in CD40 expressing tumor cells. In comparison with other CD40 agonistic antibodies such as CP-870, 893 and SGN-40 analogs, APX005M has more potent CD40 agonistic effects and antibody effector function.

